# Precision surgery in the era of 3D visualization, AR/VR, and 3D printing: current applications and future directions

**DOI:** 10.3389/fmed.2026.1688748

**Published:** 2026-03-05

**Authors:** Xiangxiang Ren, Tianhao Xie, Xiaoshi Jin, Litao Liu, Lingyun Liu, Meng Zhang

**Affiliations:** 1 Department of General Surgery, Affiliated Hospital of Hebei University, Baoding, China; 2 Department of Dermatology, Affiliated Hospital of Hebei University, Baoding, China

**Keywords:** 3D printing, 3D visualization, augmented reality, general surgery, precision surgery, virtual reality

## Abstract

The advancement of precision surgery demands increased operative accuracy, underscoring the growing importance of three-dimensional (3D) visualization technology as a key tool for overcoming the limitations of two-dimensional (2D) imaging. Over the past decade, this technology has evolved from a post-processing tool into an integrated intelligent platform spanning the entire surgical workflow: preoperative assessment, surgical planning, intraoperative navigation, postoperative follow-up, and teaching/training. This report comprehensively reviews the current applications and key advances of 3D reconstruction, 3D printing, augmented reality (AR), virtual reality (VR), and mixed reality (MR) technologies across major general surgery subspecialties. These include hepatobiliary, pancreatic, gastrointestinal, thyroid/breast, hernia/abdominal wall, organ transplantation, and pediatric surgery. Through in-depth analysis, the review elucidates how these technologies facilitate precision surgery, objectively assesses current technical limitations and ethical/regulatory challenges, and explores future directions driven by artificial intelligence (AI), 5G/6G telecommunications, and digital twin technology.

## 1 Introduction

The era of precision surgery demands unprecedented capabilities from surgeons to achieve the objectives of “seeing clearly, calculating precisely, and operating steadily.” Traditional 2D medical imaging modalities—such as computed tomography (CT), magnetic resonance imaging (MRI), and ultrasound—provide invaluable anatomical information. However, their planar display inherently leads to a loss of spatial depth perception and obscures critical relationships between adjacent tissues. Surgeons must therefore perform complex mental conversions from 2D to 3D representations, a process that increases cognitive load and introduces potential for misjudgment.

Three-dimensional (3D) visualization technology has emerged as a pivotal solution. Its evolution has progressed from early static reconstructions to dynamic interactive models, and now to sophisticated intelligent platforms integrating AI, AR, and VR. Within general surgery, applications have rapidly expanded beyond initial focuses like hepatobiliary planning to encompass the entire surgical workflow, including preoperative precision assessment, navigation for complex procedures, personalized treatment planning, postoperative follow-up, and teaching. This transformation is profoundly reshaping surgical paradigms (1).

3D visualization technology addresses the inherent limitation of 2D imaging by integrating multimodal data—such as CT, MRI, ultrasound, and positron emission tomography (PET)—and applying advanced computer graphics algorithms. It generates intuitive, volumetric, and interactive representations of complex anatomy, lesion morphology, and their spatial relationships with surrounding critical structures (e.g., vasculature, bile ducts) (2–6). This “god’s-eye view” provides a robust technical foundation for achieving personalized, minimally invasive, and function-preserving precision surgery.

## 2 Overview of key technologies

The 3D visualization platform is a sophisticated integrated system encompassing multiple cutting-edge technologies. The following sections outline the core modules and their recent progress.

### 2.1 3D reconstruction and visualization

Three-dimensional reconstruction forms the foundation, aiming to precisely segment and render 3D anatomical models from sequences of 2D images. Early techniques relied on semi-automated methods like region growing based on intensity thresholding, combined with volume or surface rendering (7). These methods were operator-dependent and demonstrated limited efficiency and accuracy for soft tissues with poorly defined boundaries.

In recent years, the introduction of deep learning, particularly convolutional neural networks (CNNs), has revolutionized image segmentation by enhancing automation and accuracy. Models like the U-Net architecture and its variants have become the *de facto* standard in medical image segmentation (8, 9). U-Net’s encoder-decoder structure with skip connections enables effective capture of multi-scale features for precise segmentation. Building on this, the nnU-Net (no-new-Net) framework further reduces application barriers by adaptively configuring optimal preprocessing, network topologies, training schemes, and postprocessing for different datasets (10, 11) (Table 1).

**Table 1:** Key performance metrics of nnU-Net in various segmentation tasks, highlighting its clinical relevance.

Segmentation task	Model/method	Dice similarity coefficient (DSC)	95% Hausdorff distance (95% HD, mm)	References	Remarks
Liver segmentation	nnU-Net	0.974 (mean)	2.458	(72)	Comparable to or exceeding expert-level performance
Specific organ	nnU-Net	Up to 0.9255 (92.55%)	Significantly reduced	(7, 73)	Performance varies by specific organ studied
Abdominal organs	nnU-Net	Typically >0.90	Significantly reduced	(7, 73, 74)	Consistent performance across organs (e.g., liver, pancreas)

Additionally, to meet demands for real-time intraoperative interaction, rendering technologies have evolved significantly. Cloud-based GPU computing and real-time ray tracing now enable smooth manipulation—including rotation, scaling, transparency adjustment, and virtual dissection—of high-quality, large-scale 3D models. These advances provide the computational foundation for complex planning and navigation (12).

### 2.2 3D printing

3D printing technology transforms digital 3D models into tangible physical models, providing a powerful platform for surgical planning, doctor-patient communication, and education. Modern multi-material, multi-color printers enable precise 1:1 replication of patient-specific anatomy. By utilizing materials with varying colors and rigidity to represent different structures (e.g., tumors, arteries, bile ducts), these models make complex spatial relationships immediately apparent (13–15). Surgeons can perform repeated simulated dissection and rehearsal on these models, optimizing strategies and anticipating risks. Beyond anatomical replicas, 3D printing shows significant potential for fabricating personalized medical devices like bioresorbable scaffolds and patient-specific implants (16–19).

### 2.3 Augmented reality, virtual reality, and mixed reality

Virtual Reality technology uses head-mounted displays (HMDs) to immerse users in computer-generated virtual surgical environments, providing an ideal platform for risk-free, repeatable skills training. In contrast, AR and MR technologies overlay virtual 3D anatomical models onto the real-world view. Surgeons wearing see-through HMDs, such as HoloLens (Microsoft) or Magic Leap, can visualize 3D models superimposed directly onto the patient’s anatomy, achieving a “radiographic perspective” effect (20, 21).

HoloLens (primarily v2) is widely adopted in healthcare, with substantial experience in neurosurgical and orthopedic navigation. Its strengths include a relatively mature ecosystem and validated medical applications (22). A primary limitation is its relatively narrow field of view (FOV) (23).

Magic Leap (One/Two) is characterized by a larger FOV and advanced light-field display technology. It offers more immersive virtual-physical integration and shows potential in applications requiring broader visual coverage. However, its ecosystem and medical application maturity are still developing compared to HoloLens (24).

A core challenge for AR/MR navigation systems is registration—the precise alignment of virtual models with the patient’s actual anatomy. By integrating tracking systems, leading platforms achieve target registration errors (TRE) at the millimeter level for static organs [e.g., ~1.3 mm in liver surgery (25)]. However, for abdominal soft tissues, displacement caused by respiratory motion and surgical manipulation remains a significant challenge, potentially increasing errors to 5–12 mm (26). These error ranges currently limit AR/MR to adjunctive roles for orientation and gross anatomical guidance rather than millimeter-precision tasks such as vessel dissection or nerve sparing. Clinical tasks that can tolerate such errors include identifying general tumor location, visualizing major vascular courses, or guiding dissection planes in well-exposed, relatively static fields.

Research into haptic and force feedback technologies is progressing to enhance immersion by enabling users to perceive tissue resistance and texture when manipulating virtual instruments, which is crucial for simulating advanced procedures (27).

### 2.4 Technology comparison and selection guidelines

Different 3D visualization technologies possess distinct advantages suited to specific clinical scenarios. Table 2 summarizes their core characteristics to guide clinical selection, aiding clinicians in choosing the most appropriate technology based on procedural needs and available resources.

**Table 2:** Comparison of key 3D visualization technologies and guidelines for clinical application.

Technology	Core advantages	Primary limitations	Typical application scenarios
3D reconstruction/visualization	• Provides intuitive 3D anatomical insights. • Enables virtual dissection, measurement, and surgical simulation. • Forms the foundation for other technologies (3D printing, AR/VR).	• High-quality segmentation can be time-intensive (complex/low-quality images). • Pure screen display lacks tactile realism. • Requires high-performance hardware for real-time rendering.	• Foundational for all applications. • Preoperative detailed assessment and planning. • Simulation of complex procedures. • Educational demonstrations (screen-based).
3D printing	• Provides tangible, physical models. • Excellent tool for doctor-patient communication and education. • Enables fabrication of customized implants/devices.	• Model production is time-consuming (hours to days). • Relatively high cost (equipment, materials). • Models cannot dynamically reflect intraoperative changes.	• Teaching and understanding complex anatomy. • Discussion and rehearsal of complex surgical plans (preoperative). • Fabrication of personalized medical devices. • Doctor-patient communication requiring physical demonstration.
Virtual reality (VR)	• Delivers fully immersive experiences. • Enables risk-free, repeatable surgical skills training. • Can simulate rare cases and complications.	• Differs from real surgical environments. • Lacks realistic haptic feedback (advanced systems under development). • May cause cybersickness in some users.	• Foundational skills training for surgical residents (suturing, knot tying, basic maneuvers). • Rehearsal of complex surgical workflows. • Immersive anatomy learning.
Augmented/mixed reality (AR/MR)	• Overlays virtual information onto the real-world/surgical field. • Provides intraoperative real-time navigation and "radiographic perspective" capability. • Enables hands-free operation (HMD).	• Registration accuracy significantly impacted by organ motion (especially soft tissue). • Device comfort, field-of-view (FOV) limitations, battery life. • High system cost; clinical validation data still accumulating.	• Intraoperative real-time navigation of anatomy (e.g., vessels, nerves, tumor location). • Surgical pathway guidance (e.g., biopsy, liver resection planes). • Remote expert collaboration and guidance. • Complex anatomy teaching (overlaid on real specimens/models).

## 3 Application in hepatobiliary surgery

**Figure 1:**
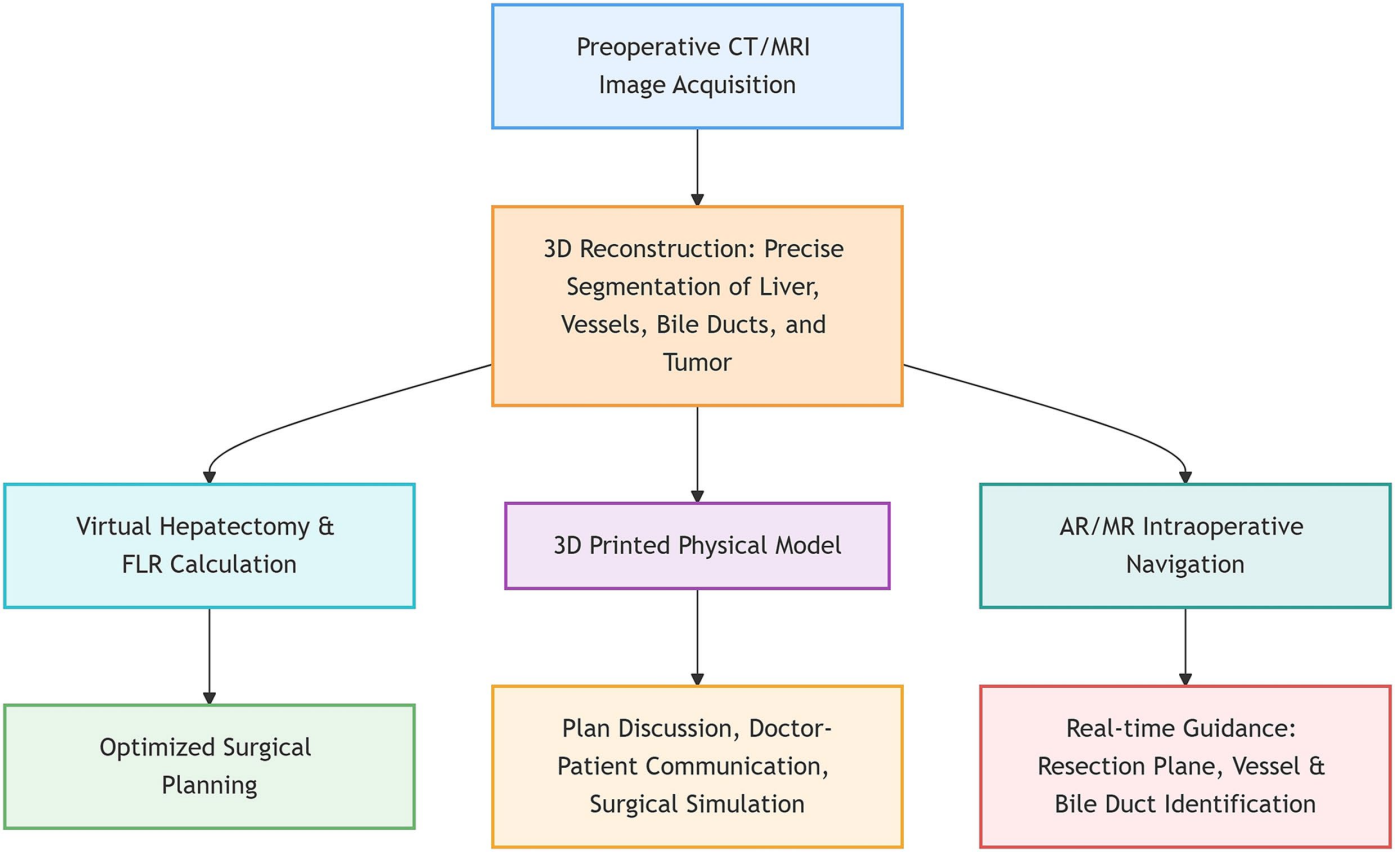
Flowchart of the application of three-dimensional visualization technology in hepatobiliary surgery.

Among general surgery subspecialties, hepatobiliary surgery represents one of the most extensively validated and impactful domains for 3D visualization technology. As shown in Figure 1, it is systematically integrated across the workflow from preoperative assessment through surgical planning and intraoperative navigation to postoperative evaluation. It enables precision segmentation of complex anatomical variants and volumetric quantification of future liver remnants (FLR), fundamentally optimizing strategy formulation for hepatectomy and biliary reconstruction.

### 3.1 Living donor liver transplantation and complex hepatectomy

In living donor hepatectomy, precise assessment of graft volume and the anatomy of vasculature and bile ducts is critical. 3D visualization provides clear delineation of vascular branching patterns, enabling optimal transection plane selection to ensure donor safety and graft viability. It plays a critical role in complex procedures like right-lobe donor hepatectomy and ALPPS (Associating Liver Partition and Portal vein ligation for Staged hepatectomy) (14, 28).

A prospective single-center study of 40 living donors demonstrated that preoperative 3D reconstruction-based virtual hepatectomy and FLR volumetry achieved a mean error of <5%, compared to 15–20% with conventional 2D CT volumetry (*p* < 0.01) (29). A multicenter study further reported that 3D surgical planning in complex ALPPS procedures substantially reduced operative time and blood loss (30). It should be noted that these improvements are based on cohort comparisons rather than randomized trials, and further validation in larger, controlled studies is warranted.

Preoperative virtual hepatectomy with FLR calculation now achieves prediction errors consistently below 5%, markedly outperforming traditional 2D methods and contributing to reduced post-hepatectomy liver failure rates (31–34). For challenging Bismuth type IV hilar cholangiocarcinomas, preoperative 3D-printed models displaying tumor invasion and perivascular relationships have become standard practice at major centers, facilitating team discussions and rehearsal (35–38).

## 4 Application in pancreatic surgery

Pancreatic surgery, particularly pancreaticoduodenectomy (PD), represents a pinnacle of technical complexity due to the organ’s retroperitoneal location and vascular proximity. 3D visualization provides critical solutions. Anatomical variations of peripancreatic vessels are highly prevalent and constitute major risk factors for hemorrhage. 3D visualization (e.g., via multiphasic CT reconstruction) achieves up to 96% sensitivity in detecting these variations, directly reducing vascular injury risk (39). It further enables intraoperative navigation with real-time anatomical registration (40, 41).

Preoperative identification of hepatic arterial variants (e.g., replaced right hepatic artery) is essential for planning vascular reconstruction (42). 3D-printed models of these complex relationships enhance trainee comprehension (43). Additionally, VR surgical simulators offer an efficient training platform for PD. Repeated rehearsal in virtual environments shortens the learning curve, with studies demonstrating reduced operative time and improved management of emergencies among VR-trained surgeons (44–46). For example, resident physicians who underwent VR training achieved 29% faster gallbladder dissection and demonstrated a 6-fold reduction in the mean number of errors committed (1.19 vs. 7.38 errors per case, *p* < 0.008, *Mann–Whitney U* test). By contrast, residents without VR training were 5 times more likely to incur gallbladder injury or thermal damage to non-target tissues (*χ^2^* = 4.27, *p* < 0.04) (45).

Although limited pancreatic mobility favors AR navigation, registration accuracy [typically 3–8 mm (26)] remains a barrier to widespread adoption. Current systems are not yet reliable for guiding precise vascular dissection but may assist in identifying major anatomical landmarks during initial exposure.

## 5 Application in gastrointestinal surgery

**Figure 2:**
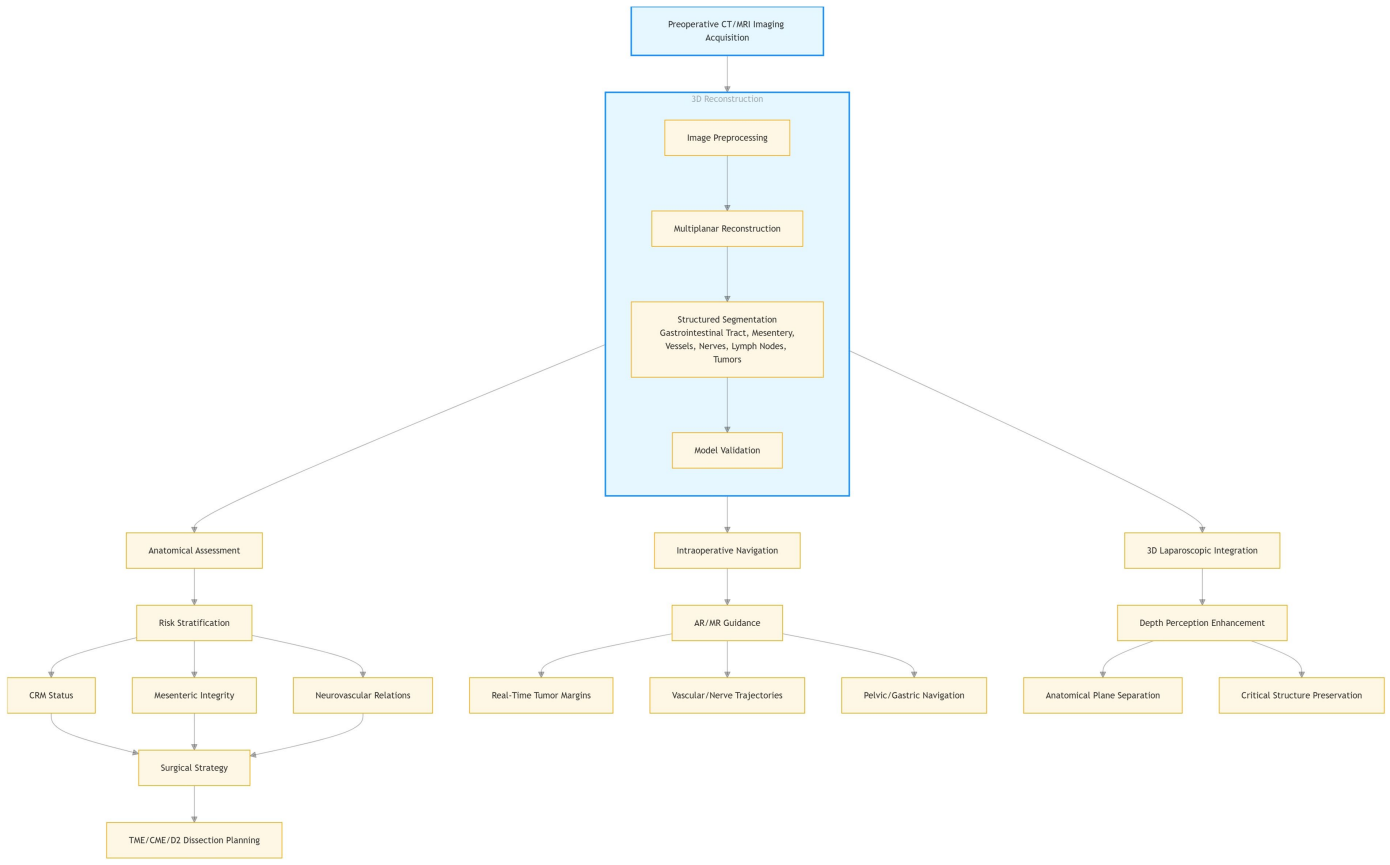
Flowchart of the application of three-dimensional visualization technology in gastrointestinal surgery.

Figure 2 illustrates the application workflow of 3D visualization technology in gastrointestinal surgery. The emphasis on *Total Mesorectal Excision (TME)* and *Complete Mesocolic Excision (CME)* principles has heightened demands for precision. While 3D laparoscopic systems provide enhanced depth perception—improving identification of planes and reducing circumferential resection margin (CRM) positivity (47)—3D visualization offers further advancements.

In low rectal cancer, preoperative 3D reconstruction of pelvic anatomy, particularly tumor relationships with pelvic walls and nerve plexuses, is critical for developing sphincter-preservation and neuroprotective strategies. During lateral lymph node dissection, AR navigation can superimpose preoperatively mapped pelvic plexus pathways onto the laparoscopic view, providing real-time avoidance guidance to preserve function while ensuring radicality (48, 49).

For gastric cancer, 3D visualization is equally valuable. D2 lymphadenectomy requires meticulous clearance around key vessels. Preoperative 3D models detect vascular variants with >95% accuracy—surpassing conventional imaging (~80%) (50, 51)—enabling anticipation of challenges. A study demonstrated 30% fewer intraoperative bleeding events and higher lymphadenectomy completeness scores with 3D surgical simulation (52, 53). However, this study was a single-center retrospective analysis (*n* = 20); prospective multicenter data are needed to confirm these benefits.

Technical Note: Clinical validation data quantifying target registration errors for AR navigation in rectal cancer [reported range: 4–7 mm (54)] remain limited. While current systems achieve errors of 1–3 mm for static structures, errors of 5–12 mm are common in abdominal soft tissues due to deformation. Such inaccuracies limit use for millimeter-precision tasks, relegating systems to adjunctive roles for orientation. For example, AR may help identify the general path of the pelvic plexus but cannot reliably guide nerve-sparing dissection at the millimeter level.

## 6 Application in thyroid and breast surgery

In thyroid cancer surgery, preserving the *recurrent laryngeal nerve (RLN)* and *parathyroid glands* defines procedural quality. Integrating preoperative 3D-reconstructed models with intraoperative indocyanine green (ICG) fluorescence imaging enables dual-modality navigation—combining anatomical mapping with real-time functional assessment. While 3D models delineate nerve trajectories, ICG visualizes parathyroid perfusion. This synergy reduces permanent RLN injury during central neck dissection to <1% (55, 56).

## 7 Application in hernia and abdominal wall surgery

Complex abdominal wall defects present significant challenges. 3D visualization enables precise preoperative quantification of defect dimensions and assessment of muscular quality, facilitating decisions regarding advanced reconstruction techniques like the Component Separation Technique (CST). Furthermore, patient-specific 3D-printed meshes, customized from CT data, achieve optimal defect adaptation, minimizing redundancy and tension to potentially reduce recurrence (57, 58). In minimally invasive repairs, AR navigation can provide real-time highlighting of key dissection planes (e.g., preperitoneal space), guiding precise separation while minimizing injury risk.

## 8 Application in pediatric general surgery

**Figure 3:**
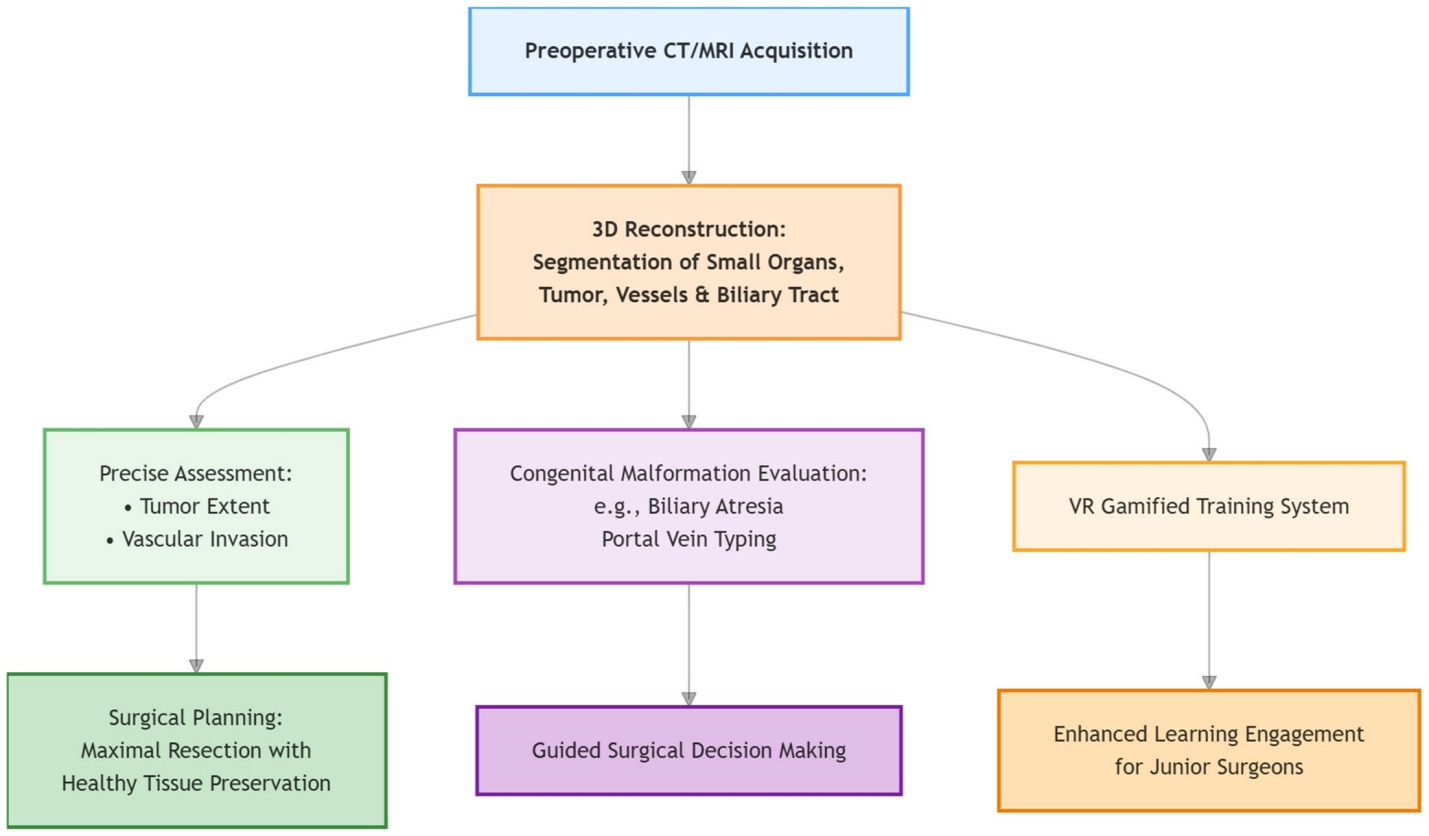
Flowchart of the application of three-dimensional visualization technology in pediatric surgery.

Figure 3 outlines the application workflow of 3D visualization technology in pediatric surgery. In this field, small organ size and intricate anatomy demand exceptional precision. For hepatoblastoma resection, 3D visualization-guided preoperative assessment and planning have become the standard to achieve maximal tumor resection while preserving functional parenchyma (59, 60).

The technology is vital in managing biliary atresia (BA), where preoperative 3D reconstruction provides stereoscopic visualization of portal vein branching patterns and spatial relationships. This delineation is critical for determining optimal dissection depth during portoenterostomy (Kasai procedure), minimizing hemorrhage risk (61).

Furthermore, VR demonstrates unique advantages in pediatric surgical education. Transforming traditional anatomy learning into gamified VR modules significantly enhances engagement among junior residents. Studies confirm a 35% improvement in anatomical knowledge test scores compared to conventional methods (*p* < 0.01) (62, 63).

## 9 Application in transplant surgery

In transplantation, 3D visualization and printing technologies primarily enhance preoperative planning and communication. For donors and recipients, 1:1 physical models of livers or kidneys enable intuitive demonstration of vascular anastomotic configurations, facilitating surgical team discussions and reportedly reducing operative time (64, 65). In split-liver transplantation, AR navigation can project predefined transection planes onto the liver surface, guiding real-time execution to minimize risks (66, 67).

## 10 Applications in teaching and training

3D visualization technology is revolutionizing surgical education. Trainees receiving 3D visualization instruction demonstrate superior mastery of anatomical knowledge and surgical skills (68–70). The results of a multicenter randomized controlled trial (RCT) are compelling: among 120 surgical residents, those trained on a VR simulator for laparoscopic cholecystectomy had a 4% intraoperative gallbladder perforation rate in their first 10 procedures, compared to 14% in the control group trained traditionally (*p* < 0.05) (71). This high-level evidence supports the integration of VR into structured training curricula.

## 11 SWOT analysis: current status and challenges

After a decade of development, 3D visualization technology has achieved significant successes but faces multi-dimensional challenges for widespread adoption. A systematic SWOT analysis follows:

### 11.1 Strengths

- Enhanced Spatial Cognition: Overcomes 2D limitations, reducing cognitive load and misjudgment risks.
- Empowered Precision Decision-Making: Enables precise preoperative assessment, personalized planning, and intraoperative navigation.
- Optimized Safety and Outcomes: Confirmed value in reducing complications, shortening operative time, and improving resection rates.
- Revolutionized Teaching and Training: VR simulators provide risk-free, immersive training; 3D/AR enhances anatomy teaching.
- Improved Doctor-Patient Communication: 3D-printed models and visualizations improve understanding and informed consent.

### 11.2 Weaknesses

- Technical Bottlenecks: Segmentation efficiency in complex cases often requires manual correction (15–45 min). Organ deformation causes registration errors of 5–12 mm in abdominal surgery, limiting precision guidance. Processing ultra-large models for real-time interaction demands high computational power.
- Clinician Acceptance and Training Gaps: Proficiency requires significant additional time and effort. Over-reliance may erode independent judgment skills. Standardized curricula and certification are not yet widespread.
- Cost and Accessibility: High costs for hardware/software, consumables, and IT support create economic barriers.
- Data Management and Interoperability: Challenges in integrating multi-modal data and poor interoperability between vendor systems.

### 11.3 Opportunities

- Deep AI Integration: For fully automated segmentation, intelligent biomechanical models predicting deformation, and AI-assisted planning.
- Next,-Gen Communication and Computing: 5G/6G and cloud computing enabling cloud-based MR and remote collaboration; edge computing for low-latency processing.
- Digital Twins and Predictive Medicine: Patient-specific “digital twins” for preoperative simulation and outcome prediction *(Current Status: Largely experimental; clinical implementation remains nascent and is supported primarily by proof-of-concept studies)*.
- Materials Science Innovations: More realistic, accessible 3D printing materials and bioprinting.
- Improving Policy and Reimbursement: Growing clinical evidence may lead to more regulatory approvals and insurance coverage.

### 11.4 Threats

- Regulatory and Ethical Challenges: AR/MR systems face complex medical device certification processes. Data security and privacy risks require compliance with GDPR, HIPAA, etc. Liability attribution for adverse decisions based on 3D models or AI remains unclear.
- Regional and Institutional Resource Disparities: Adoption is concentrated in top-tier centers, with low uptake in primary care and resource-limited settings, potentially widening the digital divide.
- Ongoing Cost-Effectiveness Pressure: High costs require robust health economic evidence to justify adoption.
- Rapid Technological Obsolescence: Fast-paced iteration risks early investments becoming outdated.

In summary, 3D visualization technology demonstrates immense strengths in general surgery but is constrained by technical bottlenecks (especially registration), training needs, high costs, regulatory hurdles, and resource disparities. Realizing its equitable value requires capitalizing on opportunities presented by AI and 5G/6G while addressing threats related to ethics, regulations, data security, and resource equity.

## 12 Future directions

Looking ahead, 3D visualization technology will evolve towards greater intelligence, real-time capability, integration, and accessibility, dependent on interdisciplinary innovation and systemic support.

### 12.1 AI-driven automation and enhanced navigation

Future developments include achieving fully automated real-time segmentation through advanced AI architectures (e.g., Transformer, graph neural networks) and self-supervised learning, enabling robust “one-click” segmentation and eliminating manual correction. It must be emphasized that while promising, these capabilities are currently experimental and require extensive clinical validation before routine use. This will be coupled with the development of multi-organ coupled deformation prediction models integrating biomechanics to sense and compensate for organ displacement in real-time. Overcoming registration inaccuracies remains a critical hurdle. Future systems must integrate real-time biomechanical modeling and intraoperative imaging to achieve dynamic registration accuracy within 2 mm, which is essential for trusting AR/MR guidance in critical dissection.

### 12.2 Cloud-based collaboration and digital twins

5G/6G networks will empower cloud-based mixed reality and remote collaboration by offloading intensive computing tasks to the cloud. This can create shared surgical spaces allowing geographically dispersed experts to collaborate in real-time. The ultimate goal is developing patient-specific “digital twins” that incorporate not only high-fidelity anatomy but also simulate physiological processes, mechanical feedback, and pathological evolution. Surgeons could conduct unlimited preoperative rehearsals on these twins to evaluate outcomes and prognosis of different strategies, enabling truly predictive precision surgery. These concepts remain largely in the research phase, with few clinical implementations to date.

### 12.3 Integrated platforms and standardization

Future platforms will evolve into unified collaborative environments seamlessly connecting surgeons, radiologists, engineers, and other specialists. These platforms will facilitate data fusion, collaborative planning, and knowledge sharing. Establishing unified technical standards and guidelines is paramount, covering data acquisition/processing, application workflows, and standardized outcome reporting. Authoritative organizations should lead the development of evidence-based clinical guidelines.

### 12.4 Health economics and sustainable adoption

Rigorous cost-effectiveness and cost-utility analyses are crucial to quantify technological inputs against comprehensive outcome benefits. Reliable health economic evidence should drive payers to explore innovative value-based payment models. Concurrently, innovations in materials science and 3D printing (e.g., biocompatible smart materials, faster printing techniques, bioprinting) will reduce barriers and expand applications.

The future of 3D visualization lies in building an integrated ecosystem encompassing AI, high-speed communication, biomechanics, multidisciplinary collaboration, standardization, and value-based assessment. Only through this holistic approach can current bottlenecks be overcome, enabling the transition from “technological innovation” to “accessible clinical value.”

## 13 Conclusion

3D visualization technology has evolved from a supplementary tool into a core platform driving the transformation of general surgery toward precision, intelligence, and personalization. It has profoundly reshaped how surgeons understand pathology, plan procedures, execute operations, and transfer expertise.

From complex hepatobiliary-pancreatic surgeries to nerve-preservation procedures in gastrointestinal and thyroid surgery, and to personalized strategies in hernia repair and transplantation, 3D visualization now permeates critical workflows across all subspecialties. It has significantly enhanced procedural safety, accuracy, and outcomes.

Nevertheless, broad clinical adoption still faces challenges, including algorithmic inefficiency, navigation inaccuracy, regulatory barriers, and high costs. It is crucial to distinguish between technologies with strong evidence and routine use (e.g., 3D printing for planning, VR for training) and those still evolving (e.g., AR/MR navigation, digital twins).

Realizing the technology’s transition *from availability to utility* and *from pilot demonstrations to widespread adoption* hinges on collaboratively addressing these bottlenecks. Continued refinement of AI-driven algorithms and breakthroughs in motion compensation are essential. Concurrently, establishing robust validation, securing regulatory approvals, implementing cost-reduction strategies, and developing standardized training frameworks are critical.

Looking forward, the deep integration of AI, next-generation communications, and digital twins holds promise to expand the scope and depth of applications. We are confident that with further evidence, validation, and standardization, this technology will ultimately forge a new surgical paradigm that bridges the virtual and physical realms, amplifying human expertise to redefine surgical care and inaugurate a patient-centered era of precision surgery.
